# Kawasaki Disease Increases the Incidence of Myopia

**DOI:** 10.1155/2017/2657913

**Published:** 2017-07-30

**Authors:** Yung-Jen Kung, Chang-Ching Wei, Liuh An Chen, Jiin Yi Chen, Ching-Yao Chang, Chao-Jen Lin, Yun-Ping Lim, Peng-Tai Tien, Hsuan-Ju Chen, Yong-San Huang, Hui-Ju Lin, Lei Wan

**Affiliations:** ^1^Department of Veterinary Medicine, National Chung Hsing University, Taichung, Taiwan; ^2^Department of Pediatrics, China Medical University Hospital, Taichung, Taiwan; ^3^College of Medicine, China Medical University, Taichung, Taiwan; ^4^Department of Ophthalmology, China Medical University Hospital, Taichung, Taiwan; ^5^Department of Biotechnology, Asia University, Taichung, Taiwan; ^6^Department of Pediatrics, Changhua Christian Children's Hospital, Changhua, Taiwan; ^7^School of Medicine, Chung Shan Medical University, Taichung, Taiwan; ^8^Department of Pharmacy, College of Pharmacy, China Medical University, Taichung, Taiwan; ^9^School of Chinese Medicine, China Medical University, Taichung, Taiwan; ^10^Management Office for Health Data, China Medical University Hospital, Taichung, Taiwan; ^11^Department of Obstetrics and Gynecology, China Medical University Hospital, Taichung, Taiwan; ^12^Research Center for Chinese Medicine & Acupuncture, China Medical University, Taichung, Taiwan

## Abstract

The prevalence of myopia has rapidly increased in recent decades and has led to a considerable global public health concern. In this study, we elucidate the relationship between Kawasaki disease (KD) and the incidence of myopia. We used Taiwan's National Health Insurance Research Database to conduct a population-based cohort study. We identified patients diagnosed with KD and individuals without KD who were selected by frequency matched based on sex, age, and the index year. The Cox proportional hazards regression model was used to estimate the hazard ratio and 95% confidence intervals for the comparison of the 2 cohorts. The log-rank test was used to test the incidence of myopia in the 2 cohorts. A total of 532 patients were included in the KD cohort and 2128 in the non-KD cohort. The risk of myopia (hazard ratio, 1.31; 95% confidence interval, 1.08–1.58; *P* < 0.01) was higher among patients with KD than among those in the non-KD cohort. The Cox proportional hazards regression model showed that irrespective of age, gender, and urbanization, Kawasaki disease was an independent risk factor for myopia. Patients with Kawasaki disease exhibited a substantially higher risk for developing myopia.

## 1. Introduction

Clinically significant refractive errors (REs) are the most common visual disorders. Myopia affects more than half of young adults worldwide [1, 2]. In Asian countries, the prevalence of myopia has approached epidemic proportions [3–5]. The prevalence of myopia has rapidly increased in recent decades and has led to a considerable global public health concern [6, 7]. Worldwide, there are approximately 153 million people aged > 5 years who suffer from visual impairments, 8 million of which were potentially blinded because of untreated myopia and other REs [8]. Patients with high myopia are at higher risk of developing macular and retinal complications.

Kawasaki disease (KD) is a systemic disease with unknown etiology [9]. It presents as systemic vasculitis of middle-sized arteries and often affects children aged < 5 years. It is a rare disease; however, Taiwan has reported a relatively high incidence (69 in 100,00 children < 5 years) [10]. Systemic inflammation in patients with KD leads to fever and progresses to involve multiple organ systems. About 20–25% of untreated patients with KD develop coronary artery lesions, such as aneurysm or ectasia [11].

There are several different hypotheses on the etiology of KD: infectious, genetic, or autoimmune disease. Recently it was thought that immune system regulation disorders played a significant role in the pathogenesis of KD. Marked immune dysregulation has been noted in acute KD, including activation of endothelial cells, monocytes/macrophages, CD8^+^ T cells, and oligoclonal immunoglobulin A plasma cells [12–15]. Patients with KD also showed increased levels of inflammatory cytokines and chemokines, including tumor necrosis factor-alpha, interleukin-1 beta, interleukin-6, interleukin-8, and monocyte chemoattractant protein-1 [16]. Abnormal immune responses induced the production of autoantibodies. Antiendothelial cell antibodies (AECAs) have been detected in patients with KD [17]. The cytotoxic activities of AECAs in patient sera were confirmed by incubating patient sera with human umbilical vein endothelial cells pretreated with interleukin-1, tumor necrosis factor-alpha, or interferon-gamma, which indicated a role of autoantibodies in the pathogenesis of vasculitis in patients with KD.

Recently, we found that there are higher incidence of myopia among patients with type 1 diabetes, uveitis, and systemic lupus erythematosus compared to those without inflammatory diseases. And the correlation between inflammation and myopia was further confirmed by form-deprivation animal model [18]. Since patients with KD develop eye symptoms, we aimed to identify whether the inflammation initiated by KD also affected myopia incidence. To reveal the influence of KD on myopia, we conducted a nationwide prospective cohort study to determine whether KD increased the risk of myopia.

## 2. Methods

### 2.1. Database

Taiwan Bureau of National Health Insurance (TBNHI) integrated all 13 public health insurance systems since 1995 into a large insurance program [19]. It is mandatory for people in Taiwan to join in this program and over 99% of the 23 million Taiwanese were covered. TBNHI entrusted National Health Research Institutes to construct and maintain the National Health Insurance Research Database (NHIRD) from this program. We obtained a set of National Health Insurance Research Database of children, in which information available was on medical records including demographic data, the dates of cares, and disease diagnoses, treatments, and costs, for the period of 1996–2008. The claims data used in this study represented information for half of all children, randomly selected from all insured population aged ≤18 years in Taiwan. The International Classification of Diseases, Ninth Revision, Clinical Modification (ICD-9-CM), was coded to identify the disease in all NHIRDs. Patient's identification code in the NHIRDs was recoded by TBNHI based on the Personal Information Protection Act. This well-documented database provides abundant materials for research. All researchers had signed the agreement for no intention to obtain patients' privacy. This study was also approved by the Research Ethics Committee in China Medical University Hospital, Taiwan (CRREC-103-048 (CR-2)).

### 2.2. Study Population, Outcome, and Comorbidity

We ascertained children aged 0 to 6 years with KD (ICD-9-code 446.1) newly diagnosed during the period from 2000 to 2004 as KD cohort, and the date for KD diagnosis was defined as index date. In Taiwan, KD is one of the 30 major categories of major illnesses (e.g., cancer, chronic mental illness, end-stage renal disease, and several autoimmune diseases) that are recognized as catastrophic illness covered under the NHI program. For each group of children with KD, 4 times the number of children without the history of KD were randomly selected from the insured children as the non-KD cohort and were frequency-matched according to sex, age (every 1-y span), and index year. Both cohorts with missing information on age and/or sex and with a previous diagnosis of myopia (ICD-9-CM: 367.1), cataract (ICD-9-code 366 and 743.3), glaucoma (ICD-9-code 365), and uveitis (ICD-9-codes 360.11, 360.12, 362.18, 363.00, 363.01, 363.03, 363.05–363.08, 363.1, 363.20, 363.21, 363.4, 364.00–364.02, 364.04, and 364.1–364.3) before index date were excluded from this study. Finally, 532 patients were included in the KD cohort and 2128 children were included in the non-KD cohort.

The primary outcome was myopia (ICD-9-CM: 367.1). All study subjects were assessed from the index date to December 31, 2008, until onset of myopia, or withdrawal from the insurance system.

The variables of relevance were sex, age (the age groups of <1, 1–3, and 4–6-years), and urbanization. Urbanization level was defined according to the NHRI report. City districts and townships in which participants were registered for insurance purposes were grouped into four levels of urbanization based on population density (persons/km^2^), population ratios of people with an educational level of college or above, of people aged over 65 years, and of agricultural workers, and number of physicians per 100,000 people [20]. Level 1 indicates the most urbanized areas; level 4 indicates the least urbanized areas.

### 2.3. Statistical Analysis

The differences between these two cohorts of sex, age group (<1, 1–3, and 4–6 years-old), and urbanization were analyzed by the Pearson chi-square test, and the mean age was analyzed by Student's *t*-test. The sex- and age-specific incidence rates (per 1000 person-y) of myopia were calculated by dividing the number of newly diagnosed myopia by the number of person-years in the KD and non-KD cohort. We used Kaplan-Meier (K-M) estimation method to depict cumulative incidence curves of myopia in cohorts with and without KD. And then we used log-rank test to examine whether these K-M curves were statistically different. The hazard ratio (HR) and 95% confidence intervals (CIs) of myopia with KD and non-KD cohorts were estimated in univariate and multivariate Cox proportional hazard regression model. The multivariate model was adjusted for gender, age, urbanization, and parents' occupational status. The log-rank test also was performed to evaluate the effect of KD on risk of myopia in different subgroup according to sex and age. SAS 9.4 statistical software (SAS Institute Inc., Cary, NC, USA) was used for all statistical analyses in this study. The significant level was set as *p* < 0.05 at two-tailed test.

## 3. Results

A total of 546 patients with KD and 2184 children without KD were included in this study.  Table 1 summarizes the baseline characteristics of the study cohorts. The mean age of the KD cohort was 2.09 ± 1.54 years and that of the non-KD cohort was 2.08 ± 1.59 years. We did not find any significant differences between sex, age, and urbanization status between the 2 cohorts (Table 1).

**Figure 1:**
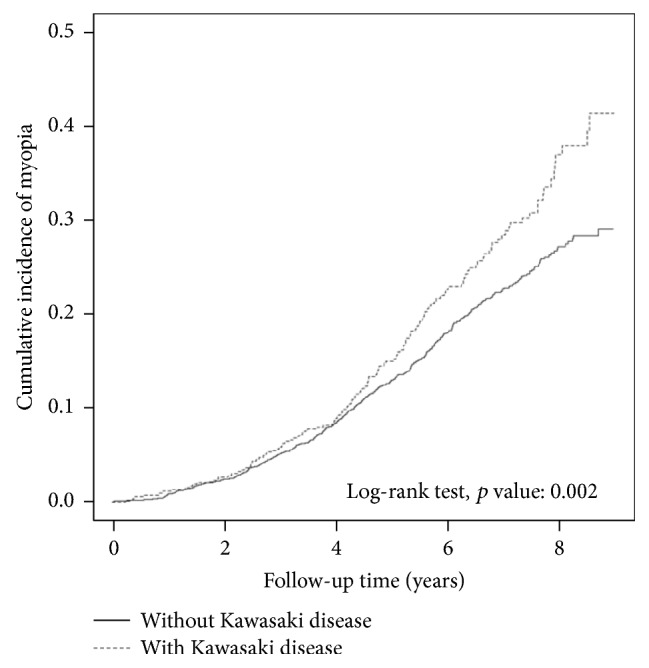
Cumulative incidence curves of myopia for with and without Kawasaki disease groups.

Figure 1 shows the cumulative incidence curves of myopia according to KD status. We used the log-rank test to examine the cumulative incidence of myopia between the cohort with and without myopia. We found that the cumulative incidence of myopia was significantly higher in the KD cohort than in the non-KD cohort (*p*  = 0.002).

Table 2 displays the results of univariate and multivariate Cox proportional hazards regression models. The adjusted HRs of myopia were significantly higher for patients with KD (HR, 1.31; 95% CI, 1.08–1.58). The risk of myopia increased with age. Patients aged 1–3 and 4–6 years exhibited 1.68- and 2.62-fold higher risk of myopia, respectively, compared with patients aged < 1 year.

A total of 425 and 137 individuals were newly diagnosed with myopia during the follow-up period in the disease cohort and control cohort, respectively (Supplementary Table  1 in Supplementary Material available online at https://doi.org/10.1155/2017/2657913). The incidence rate in the KD cohort was 43.74 per 1000 person-years and 33.38 per 1000 person-years in the non-KD cohort (Table 3). We further explored the association between KD and myopia by stratifying age, gender, and urbanization. The adjusted HRs of myopia among patients with KD indicating higher risk were observed in individuals aged 1–3 years (HR, 1.50; 95% CI, 1.17–1.91) as well as in the girl population (HR, 1.54; 95% CI, 1.14–2.08). Furthermore, we took the frequency for medical visit as the severity of KD. The data in Table 4 show that patients with KD who visited clinics more than 4 times per year exhibited a 2.66-fold increased risk of myopia after we adjusted for age, sex, and urbanization (95% CI, 1.97–3.59), compared with the non-KD cohort. The risk of myopia development increased from 0.90 (95% CI, 0.67–1.20) in patients with <1 medical visit to 1.26 (95% CI, 0.91–1.75) in patients with 2-3 medical visits and further increased to 2.66 (95% CI, 1.97–3.59) in patients with ≥4 medical visits compared with the control cohort (trend test, *p* < 0.001).

## 4. Discussion

This is the first nationwide population-based study to investigate whether patients with KD exhibited an increased risk of myopia. We excluded patients with myopia before the index date as well as those with cataract, uveitis, and glaucoma. All of the participants were assigned a unique personal identification number and could be traced through NHIRD during the study period. Compared with the non-KD cohort, risk of myopia was significantly higher for patients with KD (HR, 1.31, 95% CI, 1.08–1.58). Moreover, we demonstrated that the incidence of myopia was 43.74 per 1000 person-years among patients with KD in Taiwan. Therefore, KD increased the risk of myopia. The source of patients was based on nationwide population-based data, which covered > 99% of the population in Taiwan and decreased the selection bias.

Moreover, the longitudinal study was used to monitor myopia development between the KD and non-KD cohorts more effectively than medical chart review or cross-sectional studies. Nevertheless, there are some limitations to this study. First, diagnoses and severity of myopia cannot be quantified. Every effort was made in this large population study based on NHIRD, like in many other published studies [19]. Second, personal information, such as reading duration, light exposure time, and family history, were not available from the administrative database, and this may have compromised our results. Third, some asymptomatic or mildly symptomatic patients with myopia may not visit ophthalmologic clinics, which could lead to misclassification bias. Most myopic patients are included in the database, because it is mandatory that kindergarten, elementary, and high school students in Taiwan receive visual acuity examinations every 6 months using the logMAR chart. Those students who exhibit a visual acuity of <1.0 are asked to see an ophthalmologist to confirm the results. However, it was possible that children in the control cohort aged < 1 year in 2004 would be only 4 years of age in 2008 and therefore not of school age and hence not detected by school-based visual acuity examinations.

Ocular inflammation is a symptom commonly found in various systemic inflammatory diseases, such as rheumatoid arthritis, systemic lupus erythematosus, Sjögren's syndrome, polyarteritis nodosa, primary antiphospholipid syndrome, KD, and relapsing polychondritis [21]. The inflammatory reaction can involve different parts of the eye, including the cornea, retina, sclera, conjunctiva, and vasculature, which may result in numerous ophthalmic complications as well as potential vision impairment. Approximately 90% of patients with KD exhibited conjunctival injection, and some of the patients advanced to anterior uveitis. Myopic shift can be induced by uveitis, which can induce acute or transient myopia [22]. A 26.4-year follow-up in patients with juvenile chronic arthritis (JCA) exhibited more myopic REs compared with an age-matched control group. A total of 43% of patients with JCA had increased REs, demonstrating the association of myopia and JCA. The authors suggested that myopia progression resulted from the weakening of scleral connective tissue by chronic inflammatory responses [23, 24].

The prevalence of myopia is as high as 90% in Asian populations [25, 26]. Low-to-moderate degrees of myopia present a relatively minor inconvenience as symptoms are easily corrected by eyeglasses or contact lenses. High degrees of myopia, while also correctable using these optical aids, are of major concern because of the increased risk of myopia-related pathologies such as chorioretinal degeneration and retinal detachment [27–29]. Ocular pathologies associated with high myopia are among the leading causes of registered blindness in the developed world [27–29]. Researchers have devoted time and resources attempting to elucidate the pathogenesis, mechanisms, and treatment options for high myopia. In our previous laboratory study, we found that inflammatory reaction was closely related to the pathogenesis of myopia. In monocular form-deprivation (MFD) hamster, an experimental myopia animal model, several inflammatory related genes were expressed in the sclera and retina. The protein expression level of IL-6, TNF-*α*, c-FOS, and nuclear factor *κ*B were elevated in myopic eye and decreased upon treatment with atropine. Myopia progression was slowed down by cyclosporine A (CSA) and CSA treatment lowered the expression of IL-6, TNF-*α*, c-FOS, and nuclear factor *κ*B in the eye. While the progression of myopia was enhanced through the treatment of lipopolysaccharide (LPS) or peptidoglycan (PGN), the expression level of IL-6, TNF-*α*, c-FOS, and nuclear factor *κ*B increased. Therefore, it suggested that there was a link between inflammatory reactions and the progression of myopia [18].

In this study, we attempted to prove this theory in clinical cases. We selected a severe systemic inflammatory disease (KD) and studied its relationship to myopia. As we expected, patients with a history of KD had a higher rate of myopia. Females were more likely to have myopia than males, which has been noted in many other studies; however, no conclusion has been reached as to why more females than males develop myopia. Urbanization levels also influence the development of myopia, as it has been established that level 3 urbanization is associated with higher myopia rates; however, we found no significant differences. This may be because KD increases myopia mobility due to inflammation and not because of other environmental influences. This study suggests that eye inflammation induced by KD increases the risk of myopia incidence.

## Supplementary Material

The follow-up time of myopia associated with Kawasaki disease status stratified by age.

## Figures and Tables

**Table 1:** Demographic factors and comorbidity of study participants according to Kawasaki disease (KD) status.

Variable	No KD		KD		*p* value
	*N* = 2128		*N* = 532		
	*n*	%	*n*	%	
Gender					0.99
Girl	764	35.90	191	35.90	
Boy	1364	64.10	341	61.10	
Age, years					0.99
<1	668	31.39	167	31.39	
1–3	1192	56.02	298	56.02	
4–6	268	12.59	67	12.59	
Means (SD)	2.08	(1.59)	2.09	(1.54)	0.89
Urbanization					0.09
Level 1 (highest)	579	27.21	161	30.26	
Level 2	656	30.83	142	26.69	
Level 3	404	18.98	117	21.99	
Level 4 (lowest)	489	22.98	112	21.05	

KD, Kawasaki disease; SD, standard deviation.

**Table 2:** Cox model measured hazard ratio and 95% confidence intervals of myopia associated with Kawasaki disease and covariates.

Variables	Crude	Adjusted
	HR (95% CI)	HR (95% CI)
Kawasaki disease		
No	1.00	1.00
Yes	1.32 (1.09–1.60)^*∗∗*^	1.31 (1.08–1.58)^*∗∗*^
Gender		
Girl	1.00	1.00
Boy	0.88 (0.74–1.04)	0.90 (0.76–1.06)
Age, years		
<1	1.00	1.00
1–3	1.67 (1.36–2.04)^*∗∗∗*^	1.68 (1.37–2.06)^*∗∗∗*^
4–6	2.65 (2.04–3.43)^*∗∗∗*^	2.62 (2.02–3.40)^*∗∗∗*^
Urbanization		
Level 1 (highest)	1.16 (0.91–1.49)	1.14 (0.89–1.46)
Level 2	1.22 (0.96–1.54)	1.19 (0.94–1.51)
Level 3	1.32 (1.03–1.71)^*∗*^	1.35 (1.05–1.75)^*∗*^
Level 4 (lowest)	1.00	1.00

KD, Kawasaki disease; HR, hazard ratio; CI, confidence interval. Multivariable analyses including Kawasaki disease, gender, age, and urbanization. ^*∗*^*p* < 0.05, ^*∗∗*^*p* < 0.01, and ^*∗∗∗*^*p* < 0.001.

**Table 3:** Incidence density rates and hazard ratios for myopia according to Kawasaki disease status stratified by demographic factors.

Variables	Kawasaki disease						Compared to no KD group	
	No			Yes			Crude	Adjusted
	Event number	Person-year	IR	Event number	Person-year	IR	HR (95% CI)	HR (95% CI)
Overall	425	12730.30	33.38	137	3132.19	43.74	1.32 (1.09–1.60)^*∗∗*^	1.31 (1.08–1.58)^*∗∗*^
Gender								
Girl	158	4553.43	34.70	58	1093.06	53.06	1.56 (1.15–2.11)^*∗∗*^	1.54 (1.14–2.08)^*∗∗*^
Boy	267	8176.87	32.65	79	2039.13	38.74	1.19 (0.92–1.52)	1.17 (0.91–1.50)
Age, years								
<1	99	4310.81	22.97	30	1062.03	28.25	1.25 (0.83–1.87)	1.27 (0.84–1.91)
1–3	242	7025.93	34.44	88	1703.94	51.64	1.52 (1.19–1.94)^*∗∗∗*^	1.50 (1.17–1.91)^*∗∗*^
4–6	84	1393.56	60.28	19	366.22	51.88	0.87 (0.53–1.44)	0.87 (0.53–1.44)

KD, Kawasaki disease; IR, incidence density rates, per 1,000 person-years; HR, hazard ratio; CI, confidence interval. Adjusted HR: mutually adjusted for age, gender, and urbanization in Cox proportional hazards regression. ^*∗∗*^*p* < 0.01 and ^*∗∗∗*^*p* < 0.001.

**Table 4:** Incidence density rates and hazard ratios for myopia risk stratified by the severity of Kawasaki disease.

Average frequency for medical visit, per years	*N*	Event number	Person-years	IR	Adjusted
					HR (95% CI)
No KD cohort	2128	425	12730.30	33.38	1.00
KD cohort					
≤1	242	50	1545.08	32.36	0.90 (0.67–1.20)
2-3	163	39	976.29	39.95	1.26 (0.91–1.75)
≥4	127	48	610.82	78.85	2.66 (1.97–3.59)^*∗∗∗*^
*p* for trend					<0.001

KD, Kawasaki disease; IR, incidence density rates, per 1,000 person-years; HR, hazard ratio; CI, confidence interval. Adjusted HR: adjusted for age, gender, and urbanization in Cox proportional hazards regression. ^*∗∗∗*^*p* < 0.001.
