# Supplementary material for: Kawasaki Disease Increases the Incidence of Myopia
Source: Biomed Res Int. 2017 Jul 30;2017:2657913. doi: 10.1155/2017/2657913 (PMC5554556; doi:10.1155/2017/2657913)
Supplement: Supplementary file 1 — The follow-up time of myopia associated with Kawasaki disease status stratified by age. [file 2657913.f1.docx]

| Table S1. The follow-up time of myopia associated with Kawasaki disease status stratified by age. | | | | |
| --- | --- | --- | --- | --- |
|  | Non-KD group | | KD group | |
|  | Event no. | Follow-up, years,  Means±SD | Event no. | Follow-up, years,  Means±SD |
| Overall | 425 | 4.34±1.86 | 137 | 4.59±1.94 |
| Age, years |  |  |  |  |
| < 1 | 99 | 5.64±1.45 | 30 | 5.98±1.48 |
| 1-3 | 242 | 4.34±1.65 | 88 | 4.51±1.78 |
| 4-6 | 84 | 2.78±1.65 | 19 | 2.81±1.76 |
| Abbreviation: KD, Kawasaki disease; SD, standard deviation. | | | | |
